# Identification of Surprisingly Diverse Type IV Pili, across a Broad Range of Gram-Positive Bacteria

**DOI:** 10.1371/journal.pone.0028919

**Published:** 2011-12-21

**Authors:** Saheed Imam, Zhongqiang Chen, David S. Roos, Mechthild Pohlschröder

**Affiliations:** Department of Biology and the Graduate Program in Genomics and Computational Biology, University of Pennsylvania, Philadelphia, Pennsylvania, United States of America; Saint Louis University, United States of America

## Abstract

**Background:**

In Gram-negative bacteria, type IV pili (TFP) have long been known to play important roles in such diverse biological phenomena as surface adhesion, motility, and DNA transfer, with significant consequences for pathogenicity. More recently it became apparent that Gram-positive bacteria also express type IV pili; however, little is known about the diversity and abundance of these structures in Gram-positives. Computational tools for automated identification of type IV pilins are not currently available.

**Results:**

To assess TFP diversity in Gram-positive bacteria and facilitate pilin identification, we compiled a comprehensive list of putative Gram-positive pilins encoded by operons containing highly conserved pilus biosynthetic genes (*pilB*, *pilC*). A surprisingly large number of species were found to contain multiple TFP operons (*pil, com* and/or *tad*). The N-terminal sequences of predicted pilins were exploited to develop PilFind, a rule-based algorithm for genome-wide identification of otherwise poorly conserved type IV pilins in any species, regardless of their association with TFP biosynthetic operons (http://signalfind.org). Using PilFind to scan 53 Gram-positive genomes (encoding >187,000 proteins), we identified 286 candidate pilins, including 214 in operons containing TFP biosynthetic genes (TBG+ operons). Although trained on Gram-positive pilins, PilFind identified 55 of 58 manually curated Gram-negative pilins in TBG+ operons, as well as 53 additional pilin candidates in operons lacking biosynthetic genes in ten species (>38,000 proteins), including 27 of 29 experimentally verified pilins. False positive rates appear to be low, as PilFind predicted only four pilin candidates in eleven bacterial species (>13,000 proteins) lacking TFP biosynthetic genes.

**Conclusions:**

We have shown that Gram-positive bacteria contain a highly diverse set of type IV pili. PilFind can be an invaluable tool to study bacterial cellular processes known to involve type IV pilus-like structures. Its use in combination with other currently available computational tools should improve the accuracy of predicting the subcellular localization of bacterial proteins.

## Introduction

Type IV pili (TFP) are extremely thin, remarkably strong filaments assembled on the surface of bacterial and archaeal cells [1]. These large and varied protein assemblies are involved in a diverse array of cellular processes, including motility, conjugation, adherence, DNA uptake, and biofilm formation [1], [2], [3]. TFP are the only pili that have been identified in Gram-negative, Gram-positive, and archaeal species, suggesting an ancient origin [4], [5], [6].

**Figure 1:**
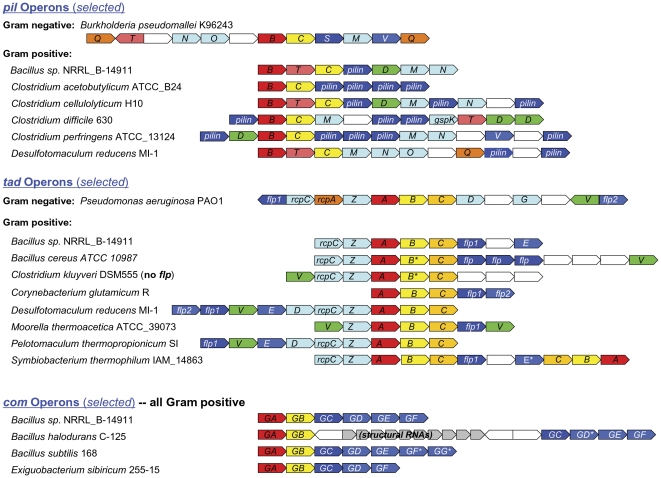
Physical maps of TFP loci in Gram-positive bacteria, compared with representative Gram-negative *pil/tad* loci. Arrows represent relative orientation of open reading frames (ORFs); genes are annotated based on high confidence BLASTP or Pfam hits against experimentally verified homologs. ORFs of the same color correspond to genes with similar function; white and gray arrows represent undefined ORFs. The *Burkholderia pseudomallei pil* and *P. aeruginosa tad* operons were selected as Gram-negative representatives based on their organizational complexity and experimentally verified components. A = *tadA*; B = *tadB* (*tad* operons) or *pilB* (*pil* operons); C = *tadC* (*tad* operons) or *pilC* (*pil* operons); D = *tadD* (*tad* operons) or *pilD* (*pil* operons); E, G, Z = *tadE*, *tadG* and *tadZ*, respectively; M, N, O, Q, S, T = *pilM*, *pilN*, *pilO*, *pilQ*, *pilS* and *pilT*, respectively; V = *tadV* (tad operons) or *pilV* (*pil* operons); GA, GB, GC, GD, GE, GF and GG = *comGA*, *comGB*, *comGC*, *comGD*, *comGE*, *comGF* and *comGG*, respectively. * indicates the absence of a prepilin peptidase cleavage site.

TFP biosynthesis has been most extensively studied in Gram-negative bacteria, where their assembly involves a well-conserved set of proteins, often encoded by a *pil* operon (Fig. 1). Assembly requires a polytopic membrane protein (PilC) which provides the base for pilus assembly, and a VirB11-like ATPase (PilB) that catalyzes polymerization of the pilin subunits. Operons containing genes that encode these core proteins may also harbor genes encoding additional biosynthetic proteins (Table 1) [2], [7], [8], including PilM and PilN (involved in the formation of an inner membrane complex needed for protein secretion), as well as PilQ (which forms a pore in the Gram-negative outer membrane, through which proteins are transported).

**Table 1:** Components encoded by TFP operons.

*TFP*	*Proteins conserved across all TFP operons*				*Pilins*		*Additional*
*System*	ATPase	TM protein	Peptidase	Secretin	Major	Minor	*TFP Proteins*
*pil*	PilB, T	PilC	PilD, U	PilQ	PilA, S	PilV	PilF, M, N, O, W*
*tad*	TadA	TadB, C	TadV	RcpA	Flp1	Flp2, TadE	RdpB*, C; TadD, G, Z
*com*	ComGA	ComGB	ComC		ComGC		ComGD, E, F, G

*specific to Gram-negative bacteria.

Operons containing TFP biosynthesis components also commonly harbor genes encoding ‘prepilins’, which contain a tripartite amino-terminal signal peptide (charged N-terminus, central hydrophobic domain, hydrophilic C-terminus) responsible for targeting these proteins to the Sec translocation pathway for secretion across the cytoplasmic membrane [9], [10]. In contrast to Sec signal peptides, which are cleaved downstream of the hydrophobic domain by signal peptidases I or II, prepilin signal peptides are processed by PilD, a prepilin peptidase that cleaves at a glycine or alanine preceding the hydrophobic stretch [1], [2], [10], [11]. The result is a hydrophobic N-terminus, usually containing a glutamate or aspartate (E/D) at position +5, part of a motif that facilitates assembly of a scaffold mediating pilus assembly [1].

**Figure 2:**
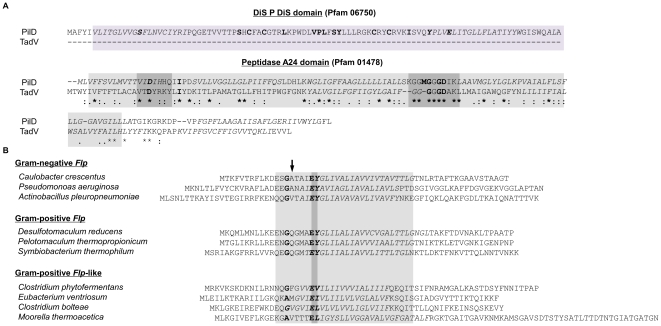
Alignment of PilD/TadV peptidases and Flp pilin signal peptides from Gram-negative and Gram-positive bacteria. (**A**) ClustalW alignment of PilD and TadV sequences from the Gram-positive bacterium *D. reducens*, including the N-terminal Dis P Dis domain (purple shading) characteristic of PilD/ComC homologs and the Peptidase A24 domain (light grey shading) found in both PilD/ComC and TadV homologs. Darker shading indicates predicted peptidase active sites, including the two essential aspartates (D). (**B**) Alignment of Gram-positive and negative pilins highlighting the Flp motif (shading), conserved TadV cleavage site (arrow), glutamate (E) at +5, and tyrosine (Y) at position +6 in Flp pilins (diverged in Flp-like pilins). Hydrophobic stretches (italics) were predicted by Phobius [28].

Flp (fimbrial low-molecular protein weight) proteins are unusually small type IV pilins (∼60 amino acids *vs.* ∼200 for *pil* or *com* pilins) that are cleaved by the truncated prepilin peptidase TadV (Fig. 2A) to expose a hydrophobic N-terminus containing a conserved tyrosine at +6, in addition to the E/D at +5, and a conserved ‘Flp motif’ within the subsequent ∼20 amino acid hydrophobic domain [1], [11], [12], [13] (Fig. 2B). Genes encoding Flp pilins are located within *tad* (*t*ight *ad*herence) loci, along with the conserved TFP biosynthetic genes *tadA*, *tadB*, *tadV* and *rcpA* (homologs of *pilB, pilC, pilD and pilQ*, respectively), and other *tad*-specific genes (*rcpB, rcpC, tadD, tadE, tadG, tadZ*) [11], [13], [14] (Fig. 1, Table 1).

In contrast to Gram-negative bacteria, most pili in Gram-positive species exploit the sortase pathway, which recognizes and cleaves a C-terminal LPXTG motif, and polymerizes pilin subunits into a macromolecular complex that is coupled to the peptidoglycan cell wall [15], [16], [17], [18], [19]. The first indication that Gram-positive bacteria possess TFP-like structures was provided by the *Bacillus subtilis* Com system, in which prepilin signal peptides are processed by the PilD homolog ComC, producing a high molecular weight DNA-binding surface structure [20], [21], [22]. In addition to the major structural subunit ComGC (encoded within the *com* operon), TFP biosynthesis requires ComGA and ComGB (homologs of PilB and PilC, respectively), but no homologs of other Pil or Tad components. Other TFP-like structures have been shown to be critical for biological processes in Gram-positives, including a *Clostridium perfringens pil* operon required for twitching motility and biofilm formation [23], [24], [25], and a *Ruminococcus albus* TFP that binds cellulose [26]. Actinobacterial operons containing three *tad*-specific genes and a *Bacillus anthracis* operon containing genes with some homology to *tad* genes have also been reported [13], [27].

Despite the importance of TFPs in both Gram-positive and Gram-negative bacteria, pilin identification can be challenging, as these genes exhibit little sequence conservation beyond the minimal motifs described above, and structural characterization is restricted by their low solubility [13], [27]. Most type IV pilin-like proteins have been identified based on association with biosynthetic genes in *pil, com* or *tad* operons, in conjunction with a potential class III signal peptidase cleavage site. However, type IV pilins need not be encoded in the same operon as biosynthetic genes [13], [27].

In order to better understand the full diversity of TFP biogenesis systems, we manually examined a representative set of Gram-positive bacterial genomes, identifying candidate pilin-encoding genes in operons that contain TFP biosynthetic genes. Gram-positive bacteria appear to express a highly diverse set of TFP operons – as many as six in some species. A subset of these putative pilins was used to define sequence features suitable for the development of a rule-based algorithm for genome-wide identification of pilin candidates. The ‘PilFind’ algorithm identified virtually all type IV pilins in a manually curated test set, with a low false positive rate. PilFind is available as an online service (http://signalfind.org) supporting genome-wide scans of user-supplied bacterial sequences, in conjunction with other signal prediction programs (Phobius, [28], TatFind [29], LipoP [30].)

## Methods

### Identification of TFP biogenesis operons and production of a type IV pilin training set

Relatively few TFP systems have been experimentally validated in Gram-positive organisms [23], [24], [25]. In order to identify new putative TFP encoding operons, predicted protein sequences for 74 completely sequenced bacterial genomes were downloaded (53 Gram-positives, 10 Gram-negatives, and 11 additional genomes lacking evidence of TFP genes; see Table S1 for sources). Putative homologs of highly conserved TFP biosynthetic proteins in Gram-positive bacterial genomes were identified using two experimentally verified VirB11-like ATPases (*B. subtilis* ComGA and *C. perfringens* PilB) and two experimentally verified polytopic membrane proteins (*B. subtilis* ComGB and *C. perfringens* PilC) as query sequences. Operons encoding proteins matching these sequences (BLASTP E-values <10^−20^) or the Pfam domains GSPII_E (found in PilB/ComGA) or GSPII_F (found in PilC/ComGB) with E-values <10^−5^ were annotated as putative TFP operons (TBG+ operons).

Genes encoding putative type IV pilins were identified in the genomes of 15 Gram-positive species (Tables 2 & S1) based on association with operons encoding the TFP biosynthetic proteins. Within these operons, genes encoding possible pilins were identified based on the presence of the canonical prepilin signal peptide motif (A/G)X_4_(D/E) [1]. A training set of 58 pilins was defined by manual curation based on the N-terminal proximity of this motif, followed by a downstream stretch of hydrophobic amino acids. Experimental evidence was also used for *B. subtilis* [21], *C. perfringens* [24], [25], *Enterococcus faecium* [31], and *Streptococcus pneumoniae* [32]. Eleven bacterial genomes lacking TFP biosynthesis genes (Table 3 & S1) were used to generate a negative training set, as these organisms are unlikely to possess type IV pilins. To facilitate the development of an algorithm capable of distinguishing type IV pilins from proteins sharing similar properties, 58 proteins were selected at random from the complement of proteins in these species containing a prepilin peptidase cleavage motif (see above), and 58 additional proteins were selected at random from the set of proteins containing a single transmembrane domain, for a total negative training set of 116 proteins (see Table S2).

**Table 2:** Gram-positive type IV pilins used to construct a positive training set for PilFind.

		TBG+ operons				PilFind+	
Species	Proteins	*pil*	*tad*	*com*	*pilins* 1	TBG+	TBG−2
***Bacillales***							
*Bacillus amyloliquefaciens* FZB42	3693	0	0	1	3	3	1
*Bacillus anthracis* str. Ames Ancestor	5611	0	1	1	4	4	1
*Bacillus licheniformis* ATCC 14580 DSM 13	4192	0	0	1	3	3	0
*Bacillus subtilis* subsp. subtilis str. 168	4105	0	0	1	3	3	1
*Geobacillus thermodenitrificans* NG80-2	3445	1	0	1	6	3	2 (2)
*Listeria monocytogenes* EGD-e	2846	0	0	1	4	4	1
*Staphylococcus aureus* subsp. aureus Mu50	2731	0	0	1	4	4	1
***Clostridia***							
*Clostridium botulinum* A str. Hall	3401	1	0	0	4	4	0
*Clostridium novyi* NT	2315	2	0	0	6	6	0
*Clostridium perfringens* str. 13	2723	2	0	0	7	7	1
*Clostridium thermocellum* ATCC 27405	3189	2	1	0	4	4	2
*Eubacterium ventriosum* ATCC 27560	2802	0	1	0	1	1	2
*Ruminococcus gnavus* ATCC 29149	3913	0	1	0	3	3	2
***Lactobacillales***							
*Enterococcus faecium DO*	2721	0	0	1	3	3	0
*Streptococcus pneumoniae* D39	1914	0	0	1	3	3	1
**Total: 15 species**	**49,601**	**8**	**4**	**9**	**58**	**58**	**13 (2)**

1 manually curated.

2 predicted pilins in operons without TFP biosynthesis genes (# PilFind positives co-transcribed with other pilins).

**Table 3:** Bacteria lacking TFP biosynthetic genes used to construct a negative training set.

		TBG+ operons				PilFind+	
Species	Proteins	*pil*	*tad*	*com*	*pilins* 1	TBG+	TBG−2
***Spirochaetales*** ** (Gram-negative)**							
*Borrelia burgdorferi* B31	1640	0	0	0	0	0	1
*Brachyspira hyodysenteriae* WA1	2644	0	0	0	0	0	1
*Brachyspira murdochii* DSM 12563	2809	0	0	0	0	0	2
*Treponema denticola* ATCC 35405	2767	0	0	0	0	0	0
*Treponema pallidum* Nichols	1036	0	0	0	0	0	0
*Treponema pallidum* SS14	1028	0	0	0	0	0	0
***Mollicutes***							
*Candidatus phytoplasma* Mali	479	0	0	0	0	0	0
*Mycoplasma genitalium* G37	475	0	0	0	0	0	0
*Mycoplasma hominis*	523	0	0	0	0	0	0
*Mycoplasma pneumoniae* M129	689	0	0	0	0	0	0
*Ureaplasma parvum* serovar 3 ATCC 27815	609	0	0	0	0	0	0
**Total: 11 species**	**14,699**	**0**	**0**	**0**	**0**	**0**	**4**

1 manually curated.

2 predicted pilins in operons without TFP biosynthesis genes.

### Analysis of TFP biosynthetic proteins and development of an algorithm for pilin identification in Gram-positive bacteria

PilB/TadA/ComGA protein sequences were aligned using ClustalX [33] (http://www.clustal.org/), and a Neighbor-Joining phylogenetic tree was constructed using the ProtDist program of the PHYLIP [34], applying 100 bootstrap pseudo-replicates to construct a consensus tree. ClustalW was used for multiple sequence alignment of the Flp subunits, and the predicted PilD and TadV peptidase domains.

The N-terminal regions of putative type IV pilins of Gram-positive bacteria from the training set were used to refine the parameters of a rule-based model (see Results). Type IV pilin motif sequences from the 58 type IV pilins in the training sets were used to construct a Hidden Markov Model (HMMER 3.0, http://hmmer.janelia.org/) in order to compare with the regular expression (RE) approach. Sequence logos were constructed using WebLogo v3 [35], and TM domains were predicted by TMHMM v2 [36] (http://www.cbs.dtu.dk/services/TMHMM/). PilFind was written in PERL programming language and is available at http://signalfind.org, where it can be used for examining user-supplied sequences.

### Assessing the performance of PilFind

In order to determine the predictive performance of the PilFind algorithm (see below) for genome-scale analysis, operons encoding TFP biosynthetic proteins were manually curated to identify probable pilins in 38 species of Gram-positive bacteria and 10 species of Gram-negative bacteria (Tables 4, 5 & S1), excluding those species used for positive and negative training data (see above, and Tables 2, 3 & S1). Recall was calculated as True Positives (TP) identified by PilFind (i.e. those that match the curated dataset), divided by the total curated dataset (i.e. TP + False Negatives; FN). The False Positive (FP) rate was assessed by using PilFind to analyze eleven genomes lacking evidence of TFP genes (Tables 3 & S1), as any hits in these species can be considered FP.

**Table 4:** Gram-positive type IV pilins identified by PilFind.

		TBG+ operons				PilFind+	
Species	Proteins	*Pil*	*tad*	*com*	*pilins* 1	TBG+	TBG−2
***Actinomycetales***							
*Corynebacterium glutamicum* R	3080	0	1	0	2	**2**	**3**
*Mycobacterium tuberculosis* H37Rv	3989	0	1	0	1	**1**	**0**
*Streptomyces avermitilis* MA-4680	7676	0	3	0	4	**4**	**2**
***Bacillales***							
*Bacillus sp.* NRRL B-14911	5691	1	2	1	7	**7**	**3 (2)**
*Bacillus cereus* ATCC 10987	5844	0	1	1	7	**7**	**2**
*Bacillus cereus* ATCC 14579	5255	0	0	1	3	**3**	**1**
*Bacillus halodurans* C-125	4066	0	0	1	0	**0**	**4 (3)**
*Bacillus thuringiensis* serovar konkukian str. 97-27	5197	0	0	1	2	**2**	**0**
*Exiguobacterium sibiricum* 255-15	3015	1	0	1	4	**4**	**2 (2)**
*Geobacillus kaustophilus* HTA426	3539	1	0	1	6	**6**	**3 (2)**
*Listeria innocua* Clip11262	3043	0	0	1	4	**4**	**2**
*Staphylococcus aureus* subsp. aureus JH1	2780	0	0	1	4	**4**	**1**
*Staphylococcus epidermidi*s RP62A	2525	0	0	1	4	**4**	**2**
***Clostridia***							
*Clostridium* sp. L2-50	2949	1	2	0	4	**3** *	**1**
*Clostridium acetobutylicum* ATCC 824	3848	1	1	0	4	**4**	**1**
*Clostridium beijerinckii* NCIMB 8052	5020	2	0	0	5	**5**	**1**
*Clostridium bolteae* ATCC BAA-613	7284	1	4	0	7	**7**	**5**
*Clostridium botulinum* A str. ATCC 3502	3590	1	0	0	3	**3**	**0**
*Clostridium cellulolyticum* H10	3390	1	3	0	4	**3** *	**0**
*Clostridium difficile* 630	3753	2	0	0	4	**4**	**4 (2)**
*Clostridium kluyver*i DSM 555	3913	2	1	0	7	**6**	**1**
*Clostridium leptum* DSM 753	3923	0	1	0	3	**2** *	**2**
*Clostridium perfringens* ATCC 13124	2876	2	0	0	9	**9**	**2**
*Clostridium perfringens* SM101	2566	2	0	0	6	**6**	**1**
*Clostridium phytofermentans* ISDg	3902	0	1	0	3	**3**	**0**
*Clostridium tetani* E88	2436	1	0	0	4	**4**	**0**
*Desulfitobacterium hafniense* Y51	5060	2	2	0	7	**7**	**1**
*Desulfotomaculum reducens* MI-1	3276	2	4	0	9	**8** *	**2**
*Halothermothrix orenii* H 168	2342	2	0	0	5	**5**	**1**
*Moorella thermoacetica* ATCC 39073	2465	1	1	0	5	**5**	**2**
*Pelotomaculum thermopropionicum* SI	2920	2	1	0	5	**5**	**1**
*Symbiobacterium thermophilum* IAM 14863	3338	0	2	0	1	**1**	**2**
*Thermoanaerobacter pseudethanolicus* ATCC 33223	2243	1	2	0	3	**3**	**0**
***Lactobacillales***							
*Enterococcus faecalis V583*	3264	0	0	1	1	**1**	**2**
*Lactococcus lactis* subsp. lactis Il1403	2321	0	0	1	3	**3**	**1**
*Lactobacillus acidophilus* NCFM	1864	0	0	1	3	**3**	**0**
*Lactobacillus brevis* ATCC 367	2218	0	0	1	4	**4**	**1**
*Streptococcus pyogenes* M1 GAS	1696	0	0	1	3	**3**	**1**
**Total: 38 species**	**138,157**	**29**	**22**	**15**	**160**	**155**	**46 (11)**

1 manually curated.

2 predicted pilins in operons without TFP biosynthesis genes (# PilFind positives co-transcribed with other pilins).

*curated pilin not identified by PilFind.

**Table 5:** Gram-negative type IV pilins identified by PilFind.

		TBG+ operons				PilFind+	
Species	Proteins	*pil*	*tad*	*com*	*pilins* 1	TBG+	TBG−2
*Actinobacillus pleuropneumoniae* L20	2012	1	1	0	5	**5**	**1**
*Burkholderia pseudomallei* K96243	5728	3	3	0	14	**12** *	**7 (3)**
*Campylobacter jejuni* subsp. jejuni NCTC 11168	1623	1	0	0	0	**0**	**6 (2)**
*Escherichia coli* ATCC 8739	4200	2	0	0	5	**5**	**3 (3)**
*Neisseria gonorrhoeae* NCCP11945	2674	1	0	0	0	**0**	**7 (3)**
*Pseudomonas aeruginosa* PAO1	5566	4	1	0	13	**13**	**9 (5)**
*Salmonella enterica* ssp enterica serovar Typhi str CT18	4758	2	0	0	2	**1** *	**6 (3)**
*Shigella dysenteriae* Sd197	4502	2	0	0	3	**3**	**4 (3)**
*Vibrio cholerae* O1 biovar El Tor str. N16961	3835	4	0	0	11	**11**	**8 (3)**
*Yersinia pestis* CO92	4066	2	1	0	5	**5**	**2 (3)**
**Total: 10 species**	**38,964**	**22**	**6**	**0**	**58**	**55**	**53 (33)**

1 manually curated.

2 predicted pilins in operons without TFP biosynthesis genes (# PilFind positives co-transcribed with other pilins).

*curated pilins not identified by PilFind.

## Results and Discussion

As a first step toward determining the diversity of TFP in Gram-positive bacteria, we selected a representative set of 53 Gram-positive bacterial genomes (Tables 2 & S1), covering all major classes of *Firmicutes* and *Actinobacteria*, for *in silico* analyses. BLASTP was used to identify homologs of the highly conserved, experimentally verified ComGA and ComGB proteins of *B. subtilis*, and PilB and PilC proteins of *C. perfringens* [22], [25], as described under Methods. The operons containing these genes were examined more closely to assess the diversity of potential TFP systems in Gram-positive species.

### Gram-positive bacteria possess a diverse array of TFP

#### Com operons

Based on the identification of putative ComGA and ComGB orthologs, all Bacillales and Lactobacillales species examined appear to harbor precisely one *com* operon (Tables 2, 4 & S1). Each of these species also contains a *pilD/comC* homolog, although not necessarily within the same operon (Table S1). No *com* operons were detected in other taxa. Most *com* operons encode a single ComGC, the major subunit of the competence surface complex (Fig. 1) [20], [21], which includes a signal peptide containing the canonical prepilin motif (A/G)X_4_(D/E) and conserved downstream sequences (displaying BLASTP E-values <10^−5^). Often, these operons also encode less well-conserved proteins containing type IV pilin-like signal peptides, which may function as minor pilins (e.g. ComGD, ComGE; [20]). In other cases, however, both major and minor pilins are encoded by operons distinct from those encoding type IV pilus biosynthetic proteins (cf. *B. halodurans*; Fig. 1).

#### Pil operons

Consistent with previous reports, many Clostridial genomes contain at least one *pil* operon similar to that found in *C. perfringens* [24], [37] that encodes homologs of *pilD*, *pilM*, and *pilN* in addition to *pilB* and *pilC* (Fig. 1). In contrast to the restriction of *com* operons to the *Bacilli*, *pil* operons were observed across a broad range of Gram-positive bacterial classes. For example, a *pil* operon was identified in *Bacillus sp. NRRL B-14911* (Fig. 1, Tables 4 & S1); this may be the first report of a *pil* operon in a *Bacillus* species.

A high degree of *pil* operon diversity was observed among Gram-positive bacteria, highlighted by variations in operon composition that may have important implications for cellular function. *pilT* (which encodes the ATPase required for pilus retraction [38]) had not previously been identified within a *pil* operon in Gram-positive bacteria, however our analysis shows that a significant number of *pil* operons contain a *pilT* homolog, indicating that these TFP may confer cellular functions requiring pilus retraction, such as twitching motility (Fig. 1 and Table S1). Intriguingly, most Gram-positive bacteria harboring *pilT* also contain a second *pil* operon that lacks *pilT*.

Despite the absence of an outer membrane, some Gram-positive *pil* operons encode a homolog of PilQ, the outer membrane secretin of Gram-negative bacteria [2] (Fig. 1). Unlike ComGC, the major pilins encoded by *pil* operons do not necessarily exhibit significant sequence conservation. All characterized *pil* operons encode at least one protein having a predicted prepilin signal peptide motif (Table S1).

#### Tad operons

Species representing most classes of Gram-positive bacteria appear to contain *tad* operons, with these operons being most widely distributed among the Clostridia. While *tad* operons previously identified in Gram-positive bacteria contain only a few of the known *tad*-specific genes (including *tadZ*, *tadC, rcpC* and *flp* [11], [27], [37]), our analysis reveals that *tad* operons of *Desulfotomaculum reducens MI-1, Pelotomaculum thermopropionicum SI*, and *Symbiobacterium thermophilum IAM 14863* contain as many as ten of the thirteen known *tad* genes (Fig. 1).

Most *tad* loci contain a gene encoding a homolog of TadV, the peptidase that cleaves Flp precursors. As in Gram-negative bacteria, the Gram-positive TadV homolog lacks much of the N-terminus transmembrane domain found in PilD (Fig. 2A), but the two aspartate residues critical for peptidase activity are evident [12]. Many operons containing *tadV* also encode putative Flp pilins. Interestingly, rather than canonical *flp* genes, some *tad* loci identified in Gram-positive bacterial genomes contain genes that encode Flp pilin-like proteins. These small proteins are similar to Flp pilins [39], but can be differentiated by the absence of a conserved tyrosine at position +6 relative to the cleavage site (Fig. 2B). Experimental evidence showing actual processing of these putative pilins will however be required. A subset of Gram-positive *tad* operons lack genes that encode either Flp pilins or Flp-like proteins, but contain a gene encoding a homolog of the TadE-like pseudopilins, suggesting that these proteins may provide the structural subunits of Tad-like pili.

A few Gram-positive *tad*-like loci lack genes that encode any protein containing an apparent prepilin signal peptide motif. Such loci, identified in the genomes of *Clostridium kluyveri, C. cellulolyticum, C. bolteae, C. sp. L2-50*, and *Desulfitobacterium hafniense*, contain homologs of several *tad* genes including *tadA*, *tadC, tadV, tadZ*, and *rcpC*, in addition to species-specific genes of unknown function (Fig. 1 & Table S1). While the *tad* gene homologs in this group of bacteria show high sequence similarity to one another, they are only distantly related to the *tad* genes of other Clostridia (with the exception of *tadA*).

#### Com, pil and tad operons form three distinct clades

**Figure 3:**
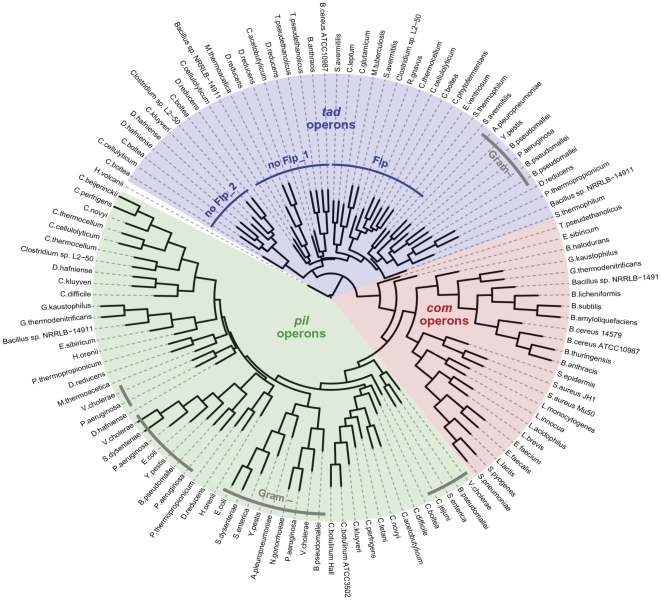
Phylogenetic classification of Gram-positive and Gram-Negative TFP operons. Phlyogenetic tree depicting the relationship between the three groups of TFP identified in Gram-positive and Gram-negative bacteria, based on PilB/ComGA/TadA homologs (using *H. volcanii* FlaI as an outgroup). Note that *pil*, *com* and *tad* operons form distinct clades, with Gram-negative bacteria grouped into clusters within each clade. Three distinct monophyletic groups can be identified within the *tad* clade, two of which encompass TFP operons that do not encode Flp pilins (no Flp_1 and no Flp_2), while the other includes all the *tad* operons encoding Flp pilins (Flp). Gram-negative sequences here highlighted in grey.

The analysis presented above clearly indicates that many Gram-positive bacteria include a combination of *pil*, *tad*, and *com* operons. As shown in Fig. 1 (and Tables 4 & S1), *D. reducens MI-1* harbors two *pil* and four *tad* operons, while *Bacillus sp. NRRL B-14911* contains one *com*, one *pil*, and two *tad* operons, making this the first species known to possess *tad, pil*, and *com* operons. While each group of TFP operons exhibits common features, the presence (or absence) of auxiliary biosynthetic genes can be used for detailed sub-classification. To further assist in categorization, we constructed a phylogenetic tree based on PilB/ComGA/TadA – one of the most highly conserved proteins encoded by TFP operons. The resulting Neighbor-Joining tree (Fig. 3) indicates that *com*, *pil* and *tad* operons form distinct clades. While the PilB and TadA sequences of Gram-negative bacteria form distinct clusters within the tree, they clearly group with their counterparts in Gram-positive bacteria. Interestingly, the PilQ-containing operons of *D. reducens MI-1*, *P. thermopropionicum SI* and *Halothermothrix orenii H 168* cluster with Gram-negative *pil* operons, while additional *pil* operons of these species group with other Gram-positive bacteria. It is possible that these PilQ encoding operons may represent instances of horizontal gene transfer from Gram-negative bacteria. Determining whether these secretin homologs are indeed part of the pilus-biosyntheis pathway will be intriguing.

Within the *tad* clade, three distinct monophyletic groups can be identified among the Gram-positive bacteria, corresponding to three groups of *tad* operons exhibiting distinct operon architecture. These include; (*i*) the *tad* operons encoding a putative Flp pilin (Flp; Fig. 3), (*ii*) operons that lack an *flp* gene but contain biosynthetic genes closely related to other *tad* operons (no Flp_1), and (*iii*) those lacking any *flp* gene, but with biosynthetic genes distantly related to other *tad* operons (see above). Overall, the classification scheme derived from phylogenetic analysis is highly consistent with that determined by examining operon composition.

### Computational identification of type IV pilins and pilin-like proteins

#### Automated identification of features associated with type IV pilin-like proteins

As noted above, most type IV pilins possess a signal peptide (including a characteristic prepilin peptidase cleavage site), harbor an N-terminal transmembrane domain, and are relatively small in size. In order to better define these features so as to facilitate prediction of type IV pilins, we manually curated a positive training set consisting of 58 type IV pilins from 15 Gram-positive bacterial genomes, including 13 experimentally verified pilins from *B. subtilis, C. perfringens, E. faecium* and *S. pneumoniae* [16]–[20], [46], [47] (see Tables 2 and S1, Methods). A negative training set was constructed from the genomes of eleven species from the Mollicutes and Spirochaetales, as these were the only class and order, respectively, that we identified as lacking TFP biosynthetic genes. In order to mimic the properties of type IV pilins, we selected 58 non-‘hypothetical’ proteins possessing one TM domain, and an additional 58 harboring a prepilin peptidase cleavage motif (see Methods, Tables 3 and S2).

**Figure 4:**
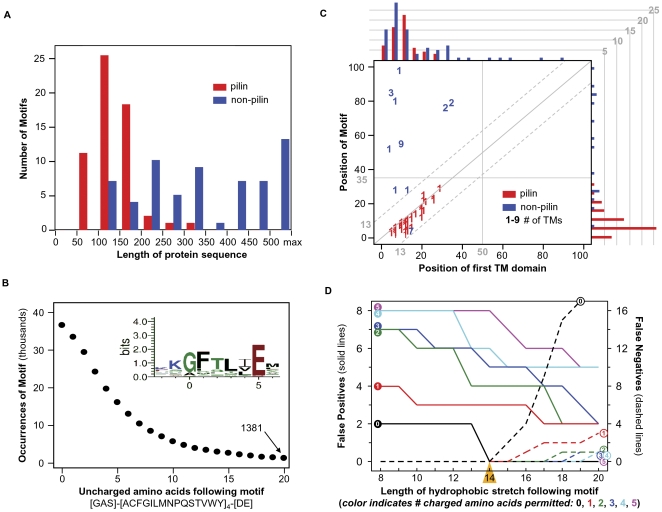
Analysis of type IV pilins features in Gram-positive bacteria training sets. (**A**) Type IV pilin sequences (red) are shorter than non-type IV pilins (blue). (**B**) Occurrence of the 6 amino acids core motif for type IV pilin peptidase cleavage, followed by a stretch of uncharged amino acids length 0–20 in 15 genomes (49,601 protein sequences; Table 2) used to define the positive training sets. ∼75% of the these proteins include the six amino acid motif [GAS]-[ACFGILMNPQSTVWY]_4_-[DE] (inset), and 1381 contain this motif followed by 20 uncharged amino acids, suggesting that many are false positives. (**C**) Relative position of the type IV pilin motif and first transmembrane domain. Histograms depict the motif position (right) and transmembrane domain position (top) for type IV pilins (red) and non-type IV pilins (blue) in the training sets, only position within 100 amino acids are shown. Note that motif distribution is relatively even in non-type IV pilins, but occurs strictly within the first 35 amino acid residues in type IV pilins. Diagonal dashed lines indicate ±13 amino acids distance between the type IV pilin motif and the first TM domain. In the scatter plot, number indicates the number of TM domains. (**D**) Assessing the effect of length and amino acid composition of the stretch subsequent to the type IV pilin cleavage pattern. Colored lines indicate the impact of permitting 0, 1, 2, 3, 4 or 5 charged amino acids within the hydrophobic stretch of length 8–20 amino acids. Solid lines represent false positives, and dashed lines false negatives. Seeking a hydrophobic stretch of 14 amino acids with no charged side chains (yellow triangle) yields optimal performance.

Analysis of these training sets define appropriate parameters for predicting type IV pilins based on these features. As shown in Fig. 4A, pilins are significantly smaller than the average protein, with a median length of 144 amino acids and maximum length of 333, as opposed to a much broader spread in the negative training set (median 430; maximum 1390). True pilins harbor the prepilin peptidase cleavage motif [GAS]-[ACFGILMNPQSTVWY]_4_-[DE], as discussed above (Fig. 4B, inset), but this motif is not sufficient to specifically identify type IV pilins: ∼73% of the predicted proteomes of the 15 species used to generate the positive training set (Table 2) include a potential cleavage motif (Fig. 4B). The specificity of pilin identification may be enhanced by considering the number of uncharged amino acids following the motif; however, 11% of the 15 species proteome still contains a prepilin peptidase cleavage motif followed by 10 hydrophobic amino acids. Specificity may be further enhanced by considering the position of the cleavage motif (usually within 35 amino acids of the N-terminus; Fig. 4C, vertical axis). While the distribution of pilin transmembrane domains is not significantly different from other transmembrane proteins (usually within 50 amino acids of the N-terminus; Fig. 4C, horizontal axis), true pilins invariably contain one TM domain only, and the position of this domain is typically close to the cleavage motif (Fig. 4C). False positive and false negative detection rates were also determined for the training set as a function of the length and hydrophobicity of amino acids immediately downstream of the cleavage motif. As shown in Fig. 4D, imposing a requirement for 14 sequential uncharged amino acids immediately following the cleavage motif yielded a perfect record in training set classification.

**Figure 5:**
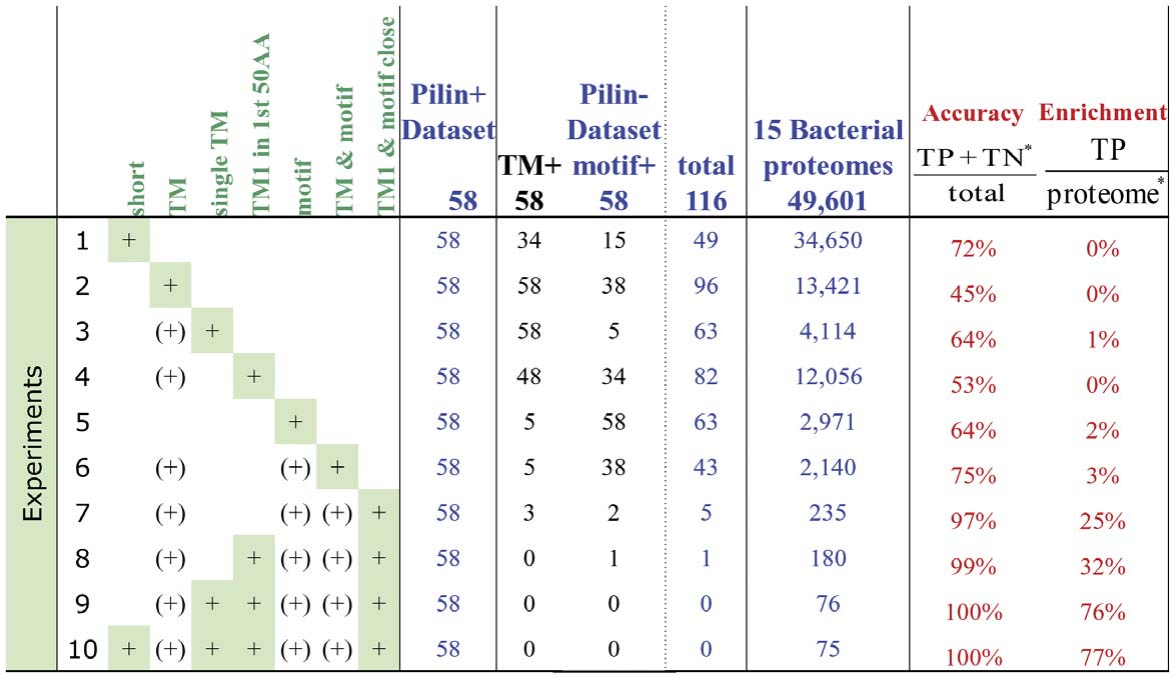
Combining features for better identification of type IV pilins. Criteria applied to the 58 protein positive training dataset, the 116 protein negative training dataset, and 15 Gram-positive bacterial genomes from which the positive training set was assembled; “Short”, protein sequence length ≤350 amino acids; “TM”, presence of a transmembrane domain, a single TM, or the first TM within the N-terminal 50 amino acids; “Motif”, presence of the characteristic prepilin peptidase cleavage recognition site followed by 14 non-charged amino acids, or in close proximity (≤13 amino acids) to the first TM. Green shading indicates criteria applied in these computational experiments. “TP”, true positive; “TN”, true negative; “proteome”, the proteins satisfying the criteria applied.

Combining these various parameters provides excellent performance in prepilin detection, as indicated for the positive and negative training set data and the 15 Gram-positive species used to assemble the positive training set (Fig. 5). Considered in isolation, protein length, the presence of a transmembrane domain, or the presence of a potential cleavage motif provide good sensitivity but poor specificity in pilin identification. Combining these parameters to search for proteins <350 amino acids in length, containing precisely one transmembrane domain within 50 amino acids of the N-terminus, and just downstream of a potential cleavage motif correctly classifies all 174 proteins in the training set (58 true positives, 58 motif+/TM− true negatives, and 58 motif−/TM+ negatives), while identifying an additional 16 candidate pilins in the genomes from which this training set was derived (Table S1).

These parameters were implemented as a PERL program entitled PilFind (available at http://signalfind.org), permitting automated searching for type IV pilin-like proteins in entire genomes (see Methods). Genome-scale performance of PilFind was evaluated against several datasets, including: (*i*) 38 representative Gram-positive genomes (Table 4), excluding the 15 genomes used in the positive training set (Table 2), (*ii*) 10 representative Gram-negative genomes, which contain a significant number of experimentally verified type IV pilins (Table 5), and (*iii*) 11 genomes lacking obvious TFP biosynthesis genes (Table 3), which were used to supply a small number of proteins for the negative training set (Table S2).

#### Performance of PilFind against Gram-positive bacterial genomes

Considering the genomes of 38 representative Gram-positive bacteria that were analyzed, PilFind identified 155 of the 160 curated putative type IV pilins within operons containing TFP biosynthesis genes (TBG+ operons), or a recall of ∼97% (Tables 4, S1 & S3). PilFind also identified an additional 57 pilin candidates outside of TBG+ operons (TBG− operons), most of which are annotated as uncharacterized hypothetical proteins. It is possible these proteins are true pilins, as in the case of *B. halodurans*, where TFP biosynthetic genes and the pilin subunits (ComGC, ComGD, ComGE and ComGF) are encoded in adjacent – but distinct – operons (Fig. 1). Operons that contain pilins without biosynthetic genes have also been reported in *Neisseria gonorrhoeae* and *Burkholderia pseudomallei* [34], [35]. In archaea, it is also not uncommon for putative pilins to be encoded by genes that are not in the same operon as homologs of TFP biosynthesis genes [4]. For example the major *Methanococcus maripaludis* pilin is neither co-regulated with known type IV pilus biosynthesis, nor with other putative pilin genes [40]. Eleven of the 57 pilin candidates in TBG− operons are encoded by genes clustered together in operons containing two or more putative pilin genes (Table 4 & S1).

#### Performance of PilFind against Gram-negative bacterial genomes

While the main intent of this work was to identify Gram-positive type IV pilins, PilFind was also evaluated against Gram-negative bacterial genomes containing core TFP biosynthesis proteins (PilB and PilC homologs). This software successfully identified 55 of 58 manually curated type IV pilins in TBG+ operons, including 16 experimentally verified type IV pilins – a recall of ∼95%. PilFind also identified 53 pilin candidates in TBG− operons, including 11 experimentally verified type IV pilins. The remaining 42 type IV pilin candidates include both hypotheticals and proteins annotated as putative pilins, although experimental verification of their true functions is lacking at present. Overall, PilFind was able to identify 27 of 29 experimentally verified type IV pilins, regardless of whether they are located within or outside of operons containing TFP biosynthesis genes, highlighting the predictive potential of this software [12], [41], [42], [43], [44], [45], [46], [47], [48], [49], [50], [51], [52], [53], [54], [55], [56], [57] (Tables 5, S1 & S3).

#### Performance of PilFind in bacteria lacking TFP-biosynthesis genes

Assessing the specificity of PilFind poses a challenge, as our catalogue of manually curated type IV pilins are almost completely restricted to TBG+ operons, making it difficult to categorize newly identified pilin candidates in TBG− operons. The observation that a significant number of experimentally verified Gram-negative type IV pilins reside in TBG− operons (see above) suggests that many of these candidates may be genuine type IV pilins. In order to assess the specificity of PilFind, we analyzed 11 additional bacterial genomes from the orders Mollicutes and Spirochaetales, as discussed above (Table 3). To the best of our knowledge, these bacteria are entirely lacking core TFP biogenesis genes, and are therefore not expected to encode type IV pilin-like proteins. PilFind identified only four pilin candidates (probable false positives) from among the 14,699 proteins predicted from these genomes, yielding a false positive rate of ∼2.7×10^−4^, or one false positive identified for every 3675 proteins analyzed – approximately the size of the entire proteome for many bacteria (Table 3 & S3).

#### Comparing PilFind's regular expression-based predictions to those obtained from a hidded Markov model

**Figure 6:**
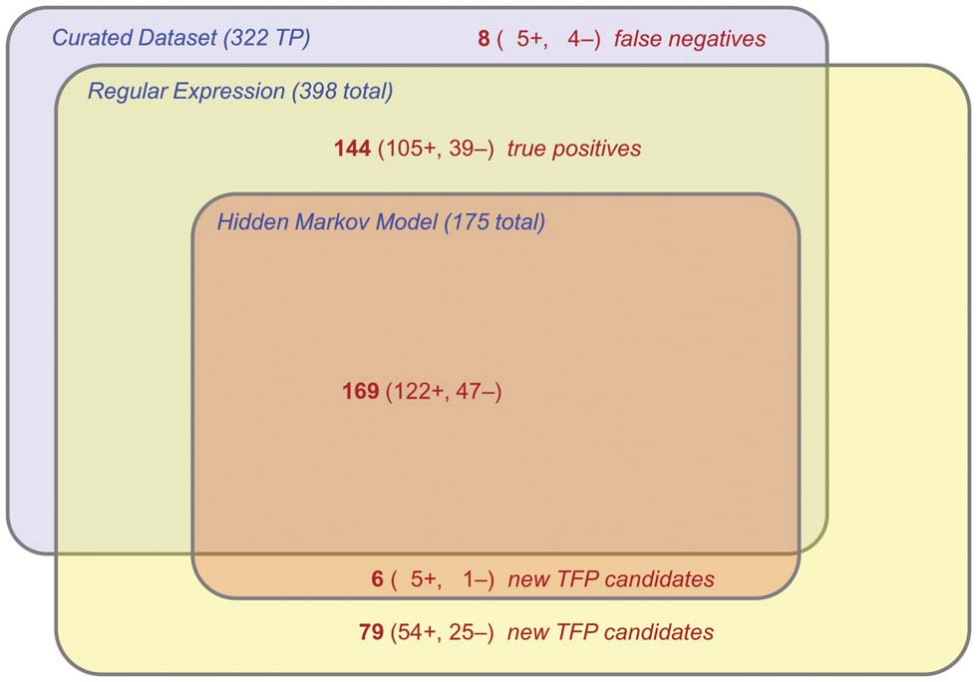
Comparison of type IV pilin predictions using Hidden Markov Model (HMM)- and regular-expression (RE)-based approaches. Venn diagram indicating the total number of type IV pilins predicted in 48 genomes (38 Gram-positive and 10 Gram-negative) using an HMM, a RE, or manual curation. The HMM approach displays high specificity, but is only capable of identifying a relatively small subset of the curated type IV pilins (106 of 218), and 24 new pilin candidates. The RE-based approach identified a much larger number of curated type IV pilin (210 of 218), along with 110 new pilin candidates, including all those of identified by the HMM-based approach, respectively. Numbers in parenthesis indicate data for the True Positive “+”, and False Negatives “−”.

PilFind performance could potentially be improved by using more sophisticated machine learning algorithms to model the type IV pilin motif, rather than a regular expression that does not consider distribution of amino acids at each position in the motif. However, given the relatively small number of validated TFP sequences for training, it is challenging to develop an algorithm that would be applicable on a genomic-scale. A profile-HMM was generated based on the same 58 protein manually-curated positive training set used to generate the regular expression described above. Applying this HMM-based approach to all 48 organisms used to test PilFind performance (38 Gram-positives, 10 Gram-negatives) identified only 106 of 218 manually curated type IV pilins (recall ∼49%), with only 24 new candidates being identified (Fig. 6). In contrast, the regular expression strategy identified 210 of 218 manually curated type IV pilins (recall ∼96%), plus an additional 110 new candidates (Fig. 6). Compared to the HMM-based approach, RE is more permissive, allowing for the identification of more pilins with only a slight decrease in specificity. Thus, the RE-based PilFind method is particularly suitable for identifying type IV pilins on a genomic-scale.

### Concluding remarks

This study identifies a highly diverse range of putative Gram-positive type IV pilins. Because many type IV pilins are subunits of cell surface structures known to play critical roles in conjugation, surface motility, biofilm formation, and other important biological activities, characterizing these Gram-positive bacterial cell surface structures is likely to greatly enhance our understanding of important cellular processes in Gram-positive bacteria. Furthermore, as type IV pili play important roles in the pathogenesis of many organisms, studying these structures may help to identify new therapeutic targets. The overall diversity of the structural subunits and biosynthetic pathway components suggests the possibility of designed therapeutics targeted to particular pathogens.

Identifying which differences in the biosynthetic machineries of Gram-negative and Gram-positive bacteria are responsible for their physiological differences may also provide useful insights into bacterial evolution. For example, we have identified Gram-positive homologs of PilQ, the secretin involved in the transport of pilins across the outer membrane of Gram-negative bacteria. Comparing the similarities and differences of TFP and their biosynthetic machineries, among and between Gram-positive bacteria, Gram-negative bacteria and Archaea, will surely provide a better understanding of how these ancient prokaryotic surface structures have come to play such important roles in diverse cellular processes. As an automated on-line server, PilFind will be valuable for such studies, as it can readily identify genes that encode pilin candidates in any given bacterial genome, similar to the previously designed program, FlaFind, trained to specifically identify archaeal pilins, most of which contain distinct prepilin peptidase processing sites. Moreover, considering the observed abundance of pilins in many bacteria, the incorporation of PilFind and FlaFind into a suite of programs predicting subcellular localization of proteins should significantly enhance accurate annotation of open reading frames encoding proteins of the secreted proteome. Finally, considering the fact that bacterial pathogenesis often involves type IV pili, PilFind provides an invaluable tool for identifying candidate virulence factors among the thousands of proteins encoded by any given pathogen genome.

## Supporting Information

Table S1 **Type IV pilus biogenesis and and pilin-like genes (with PilFind training data, GI numbers, URLs, etc).**
(XLS)

Table S2 **Negative training set used in development of PilFind.**
(XLS)

Table S3 **Features of false negatives and false positives from PilFind analysis.**
(XLS)
